# Niche-Independent Symmetrical Self-Renewal of a Mammalian Tissue Stem Cell

**DOI:** 10.1371/journal.pbio.0030283

**Published:** 2005-08-16

**Authors:** Luciano Conti, Steven M Pollard, Thorsten Gorba, Erika Reitano, Mauro Toselli, Gerardo Biella, Yirui Sun, Sveva Sanzone, Qi-Long Ying, Elena Cattaneo, Austin Smith

**Affiliations:** **1**Institute for Stem Cell Research, University of Edinburgh, Edinburgh, United Kingdom,; **2**Department of Pharmacological Sciences and Center of Excellence on Neurodegenerative Diseases, University of Milan, Milan, Italy,; **3**Institute of Physiological and Pharmacological Sciences, University of Pavia, Pavia, Italy; National Institute for Medical ResearchUnited Kingdom

## Abstract

Pluripotent mouse embryonic stem (ES) cells multiply in simple monoculture by symmetrical divisions. In vivo, however, stem cells are generally thought to depend on specialised cellular microenvironments and to undergo predominantly asymmetric divisions. Ex vivo expansion of pure populations of tissue stem cells has proven elusive. Neural progenitor cells are propagated in combination with differentiating progeny in floating clusters called neurospheres. The proportion of stem cells in neurospheres is low, however, and they cannot be directly observed or interrogated. Here we demonstrate that the complex neurosphere environment is dispensable for stem cell maintenance, and that the combination of fibroblast growth factor 2 (FGF-2) and epidermal growth factor (EGF) is sufficient for derivation and continuous expansion by symmetrical division of pure cultures of neural stem (NS) cells. NS cells were derived first from mouse ES cells. Neural lineage induction was followed by growth factor addition in basal culture media. In the presence of only EGF and FGF-2, resulting NS cells proliferate continuously, are diploid, and clonogenic. After prolonged expansion, they remain able to differentiate efficiently into neurons and astrocytes in vitro and upon transplantation into the adult brain. Colonies generated from single NS cells all produce neurons upon growth factor withdrawal. NS cells uniformly express morphological, cell biological, and molecular features of radial glia, developmental precursors of neurons and glia. Consistent with this profile, adherent NS cell lines can readily be established from foetal mouse brain. Similar NS cells can be generated from human ES cells and human foetal brain. The extrinsic factors EGF plus FGF-2 are sufficient to sustain pure symmetrical self-renewing divisions of NS cells. The resultant cultures constitute the first known example of tissue-specific stem cells that can be propagated without accompanying differentiation. These homogenous cultures will enable delineation of molecular mechanisms that define a tissue-specific stem cell and allow direct comparison with pluripotent ES cells.

## Abstract 2

Austin Smith and colleagues derive neural stem cells from mouse embryonic stem cells and demonstrate their long-term propagation in vitro.

## Introduction

Stem cells are capable of generating identical progeny through unlimited numbers of cell divisions whilst retaining the ability to respond to physiological demands by producing daughters committed to differentiate. In vivo, stem cells are thought to reside in specific cellular microenvironments, or niches, that constitute privileged settings for support of self-renewal [1–4]. In tissues that utilise stem cells to sustain cell turnover, the stem cell compartment must be renewed in balance with the production of transit-amplifying progenitors [5]. This requires either equivalence between symmetrical self-renewal and commitment divisions, or an asymmetric mode of stem cell division. Expansion of stem cells, in vivo or in vitro, unambiguously requires symmetrical self-renewal. However, with the notable exception of embryonic stem (ES) cells, it has proven extremely problematic to propagate homogenous cultures of stem cells ex vivo. Epidermal stem cells [6] and neural stem cells [7] can be expanded in vitro, although accompanied by differentiation. It is unclear whether this reflects a dependence of tissue stem cells on a cellular niche, an intrinsic bias of tissue stem cells towards asymmetric division, or a failure to develop appropriate culture conditions to suppress commitment and sustain symmetrical self-renewal, as has been achieved for ES cells [8].

Neural stem cells appear to be sustained in a complex niche in the mammalian brain [9–11]. In 1992, Weiss and Reynolds made the landmark discovery that neural stem cells could be maintained in culture via propagation of floating cell clusters termed “neurospheres” [7]. Neurospheres consist predominantly of committed progenitors mixed with differentiated astrocytes and neurons. This mixed cellular environment likely provides a niche that sustains relatively few stem cells [12]. The neurosphere assay has proven invaluable in demonstrating the potential to give rise to stem cells in the developing and adult central nervous system (CNS) of rodents and primates [13–15]. However, neurospheres have significant limitations. The stem cells maintained within neurospheres are not directly identifiable, have not been purified, and have an uncertain relationship to CNS precursor cells in vivo [16]. Cellular complexity is a barrier to molecular and biochemical dissection of self-renewal and commitment mechanisms [17]. Heterogeneity also undermines comparative analytical approaches such as global expression profiling [16]. Furthermore, there is variation between as well as within cultures, which can give rise to contradictory data from different laboratories [18]. Finally, neurospheres differentiate much more readily into astrocytes than neurons in vitro [18] and in vivo [19], providing little enthusiasm for pharmacological screening or therapeutic applications [20]. Neural progenitor cells are also propagated in adherent cultures supported by fibroblast growth factor 2 (FGF-2) [21,22], but without genetic transformation [23,24], neuronal differentiation potential is usually progressively lost in these conditions [25,26]. As in *Drosophila,* mammalian neural progenitor cells may undergo asymmetric divisions in vivo [27,28] and in vitro [29]. However, the incidence of asymmetric versus symmetric division in true stem cells, either in vivo or in neurospheres, is unknown. Here we have investigated the potential for symmetrical self-renewal of neural stem cells and maintenance of neuronal differentiation capacity in fully defined adherent cultures.

## Results

### Derivation of Self-Renewing Adherent Neural Stem Cells from ES Cells

Mouse ES cells differentiate efficiently into neural precursor cells upon withdrawal of serum in adherent monolayer culture [8] or via treatment of embryoid bodies with retinoic acid [30,31]. These precursors have previously been expanded in FGF-2 with transient retention of neuronal differentiation potential [31,32], but continuous propagation is accompanied by restriction to glial fates ([33] and unpublished data). A similar switch from neurogenic to gliogenic differentiation is consistently observed in adherent cultures of primary foetal progenitors and is suggested to recapitulate the developmental progression during formation of the nervous system [25,34].

We induced neural precursor differentiation from ES cells in serum-free adherent monoculture [35,36]. After 7 d, cells were re-plated in basal medium (NS-A plus N2) in the presence of either FGF-2 alone or FGF-2 plus epidermal growth factor (EGF). Importantly, NS-A media does not support propagation of residual undifferentiated ES cells and they are thereby eliminated from the cultures. Neural precursors initially associate into floating clusters in this media. After 3–5 d, these aggregates were harvested, separating them from any adherent differentiated cells, and re-plated in fresh medium. They attached within 2–3 d and outgrew a population of bipolar cells. Upon passaging, these cells did not persist in FGF-2 alone, but in FGF-2 plus EGF they proliferated in the absence of other cell types. These bipolar cells, named LC-1, can be continuously and rapidly propagated with a doubling time of approximately 24 h.

**Figure 1:**
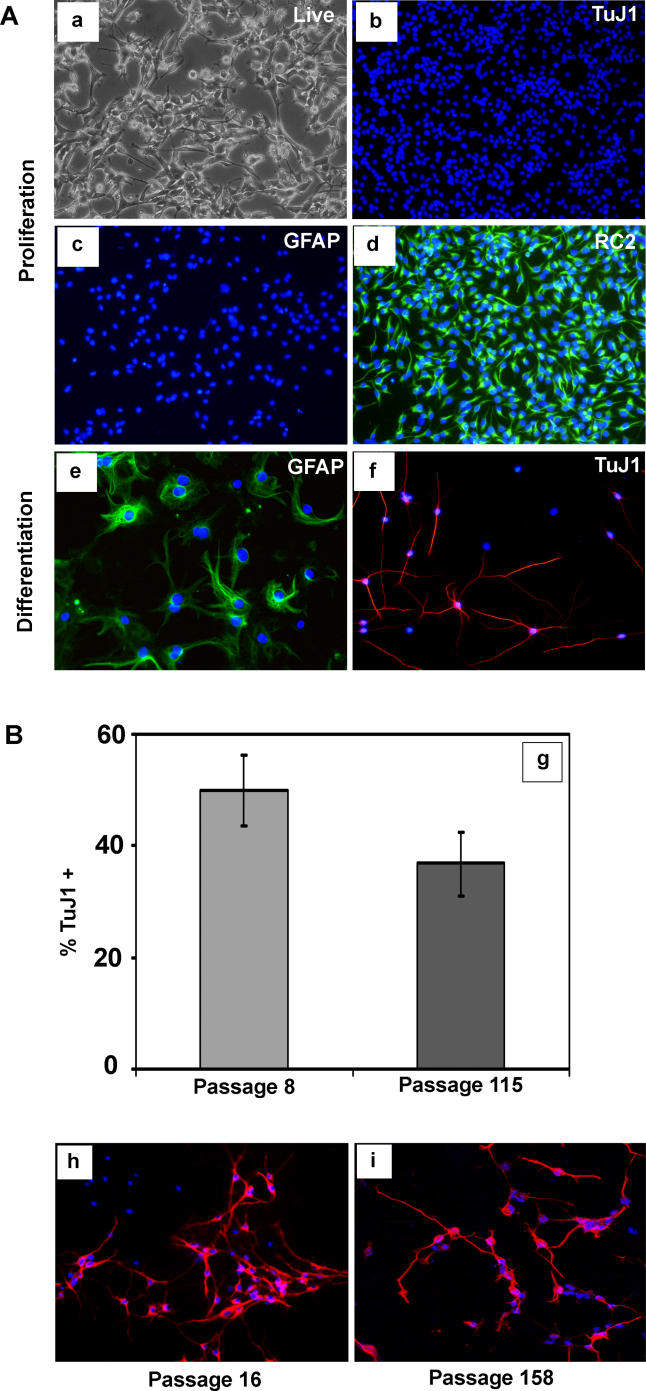
Generation of NS Cells from ES Cells (A) The adherent NS cell culture (LC1) propagated in EGF and FGF-2 (a), shows no expression of neuronal (b) or astrocyte (c) antigens, and uniform expression of the precursor marker RC2 (d) and nestin (not shown). LC1 cells differentiate into GFAP immunopositive astrocytes (e) upon addition of serum and generate TuJ1 immunopositive neurons (f) upon growth factor withdrawal. (B) The proportion of neurons obtained remains greater than 35% of total cells after 115 passages (g). Immunostaining for MAP2 of LC1 differentiation shown at passage 16 (h) and passage 158 (i).

LC1 cells express the immature neural marker nestin and are immunoreactive with the RC2 antibody, which recognises neural precursors, but expression of the astrocyte differentiation marker glial fibrillary acidic protein (GFAP) or of neuronal antigens is negligible (Parts b, c, and d in Figure 1A). Upon exposure to serum or BMP 4, LC1 cells adopt astrocyte morphology within 48 h and subsequently uniformly express GFAP (Part e in Figure 1A). In contrast, cells with fine extended processes appear after re-plating on laminin without EGF for 5–7 d and then withdrawing FGF-2. These cells express neuronal markers type III β-tubulin (Part f in Figure 1A), microtubule associated protein-2 (MAP2) (Figure 1B), and neuN (not shown). High numbers of such neuronal cells, between 30% and 40% of total surviving cells, are generated with no significant decline even after 115 passages (Part g in Figure 1B). Together with the observation that LC1 cells retain diploid chromosome content at late passages (unpublished data), these data suggest the presence of self-renewing neural stem (NS) cell cultures.

NS cells competent for both glial and neuronal differentiation have subsequently been obtained using the procedure described for LC1 from more than ten ES cell lines, originating from three independent derivations: E14, CGR8, and R1. For all NS lines examined more than 95% of cells express nestin and are immunoreactive with RC2 in the presence of FGF-2 plus EGF. These NS cells are transfectable by electroporation (unpublished data) and can reliably be recovered from standard cryopreservation.

To assess whether the serum-free adherent neural induction protocol is a prerequisite for NS cell generation, ES cells were induced to differentiate by embryoid body formation and exposure to retinoic acid in serum-containing medium [30]. Aggregates were subjected to Sox2-βgeo lineage selection with G418 [31,37] for 48 h to enrich for neural precursors, then dissociated and cultured in the presence of FGF-2 and EGF without serum. Floating clusters formed that subsequently attached and outgrew Sox2-positive, nestin-positive, proliferative cells, which displayed the bipolar morphology and lattice growth typical of NS cells, as well as the capacity for astrocyte and neuronal differentiation after multiple passages (unpublished data).

### Clonogenic NS Cells Derive from Sox1-Positive Pan-Neural Precursors

**Figure 2:**
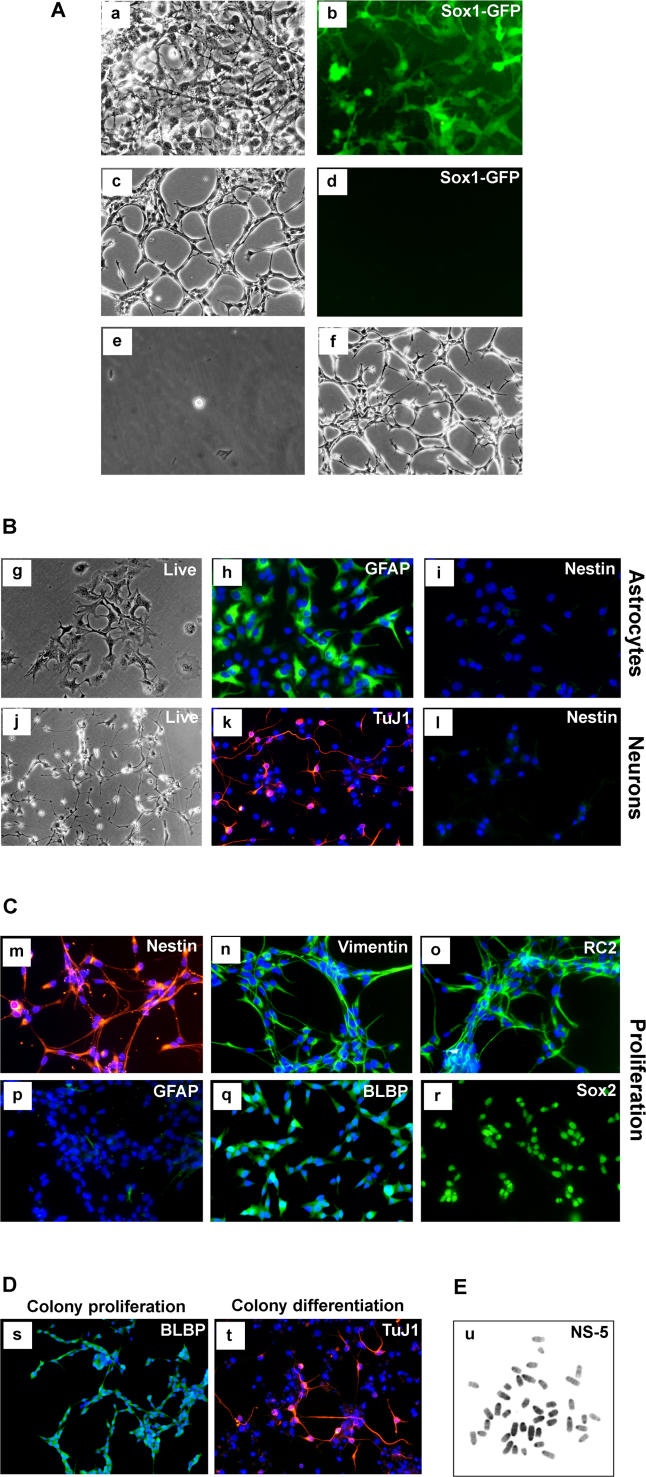
Clonal NS Cells Generated through *Sox1* Neural Lineage Selection (A) Phase image of neural precursors at passage 1 (a) and 5 (c), with (b) and (d) showing corresponding *Sox1*-GFP fluorescence. Image (e) shows a single cell, 1 h after plating in Terasaki well, and (f) shows a phase-contrast image of clonal cell line at passage 20. (B) Differentiation of NS-5 cells into astrocytes (g,h) and neurons (j,k) with loss of nestin immunoreactivity (i,l). (C) These NS-5 cells are immunoreactive for neural precursor cell/radial glia markers (m–o,q,r) and negative for GFAP (p). (D) Clones of NS-5 cells exhibit homogenous expression of BLBP with no immunoreactivity for GFAP in the presence of EGF/FGF (s), and generate neurons upon growth factor withdrawal (t). (E) Metaphase spread of NS-5 (passage 31).

To investigate the origin of NS cells and to determine whether the floating cluster phase is essential for their generation, we induced neural commitment in monolayer and then maintained the neural precursors in N2B27 medium, in which condition they remain adherent [38]. To eliminate undifferentiated ES cells and non-neural differentiation products under these conditions, we again exploited lineage selection [31]. In this case we used 46C ES cells in which the green fluorescent protein (GFP)irespac reporter/selection cassette is integrated into the *Sox1* gene, an early marker of neural specification [39]. Transient puromycin selection after differentiation induction yields a purified population of neural precursors with minimal residual ES cells [40] (Parts a and b in Figure 2A). Either FGF-2 alone or FGF-2 plus EGF were then applied to the Sox1-expressing neural precursors maintained in N2B27 medium. Appreciable numbers of bipolar cells of NS morphology appeared only in the presence of both factors (Part c in Figure 2A). Initial heterogeneity of the population reduced with two to three passages. as astrocytes and other cell types decreased in number. Notably, expression of Sox1-GFP (and endogenous Sox1) is lost during this process (Parts c and d in Figure 2A), but the cells remain positive for Sox2 and nestin. As they began to dominate the cultures, the bipolar cells formed extensive lattices. To establish the presence of clonogenic NS cells, single cells were isolated in microwells and expanded as adherent cultures (Parts e and f in Figure 2A). Five clonal lines were derived with morphology and growth characteristics similar to the bulk population. Initially one clone, NS-5, was characterised in detail, but subsequently all essential features described below were confirmed for other clones. These cells lack detectable expression of the pluripotency factors Oct4 and Nanog, and also of the early neural marker Sox1, but retain the pan-neuroepithelial marker Sox2 (Part r in Figure 2C). They are competent for astrocyte and neuronal differentiation (Figure 2B). Like LC1 cells, NS-5 cells uniformly express nestin, RC2, and other neural precursor markers (Figure 2C), and lack detectable GFAP expression (Part p in Figure 2C). We conclude that NS cells can be generated through a transient Sox1-positive neuroectodermal precursor via continuous adherent culture.

To establish that the NS-5 clone can generate sub-clones of the same phenotype, cells were plated at clonal density in EGF plus FGF-2. Multiple colonies were generated, all consisting of bipolar cells. Every colony shows immunoreactivity with RC2 (not shown) and expression of brain lipid binding protein (BLBP) (Figure 2D) in virtually all cells, with no detectable GFAP (Part s in Figure 2D). Several colonies were picked and expanded over ten or more passages with retention of these characteristics. Every colony can also be induced to differentiate into neurons (Part t in Figure 2D). Finally, we prepared metaphase spreads from NS-5 and determined a modal chromosome count of 40 (Figure 2E). The NS-5 clone therefore represents a clonogenic, genetically stable, NS cell line that self-renews continuously without requirement for a specialised cellular niche.

### Contributions of EGF and FGF to NS Cell Self-Renewal and Lineage Commitment

**Figure 3:**
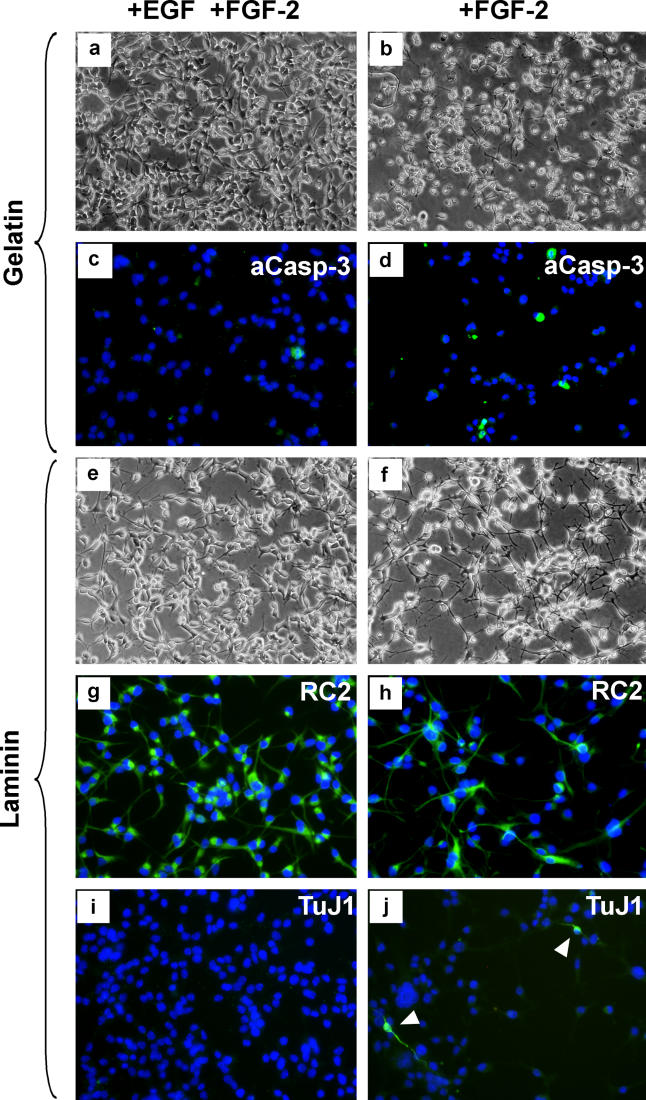
NS Cells Die or Begin to Differentiate in the Absence of EGF Unlike proliferating cultures in FGF plus EGF (A,C), NS cells on gelatin die by caspase-3-mediated programmed cell death 20 h after removal of EGF (B,D). This death can be overcome if cells are cultured on a laminin substrate in FGF-2 only (F). Under these conditions, cells become slow-dividing and extend longer processes (G,H). Most cells retain RC2 immunoreactivity (H), but a minority begin neuronal differentiation marked by TuJ1 expression (J).

As outlined above, NS cells were derived by culture only in the combination of FGF-2 plus EGF. This contrasts with previous studies, including our own, which used FGF-2 alone, resulting, after several passages, in populations of glial restricted progenitors [31–33]. We examined whether NS cells remained continuously dependent on EGF. When EGF is withdrawn from the cultures, massive cell death ensues after 20 h (Figures 3A and 3B**)**, and the few cells that survive adopt differentiated morphology. This cell death is associated with presence of activated caspase 3, indicative of apoptosis (Figures 3C and 3D). Serum or BMP override the cell death response and drive the NS cells into astrocytic differentiation. We conclude that EGF firstly allows the maintenance of stem cells with competence for neurogenesis in contrast to FGF-2 alone, and secondly supports self-renewal of NS cells, acting in part via suppression of apoptosis.

We found that laminin could preserve cell viability in the absence of EGF. Simultaneous removal of EGF and FGF-2 on laminin results in astrocyte differentiation (unpublished data). However, NS cells cultured on laminin with FGF-2 alone do not express GFAP. Instead, they develop more extended processes (Figures 3E and 3F), and slow their rate of cell division. To date, we have been unable to maintain proliferation upon dissociation and passaging using FGF-2, and the cells die out when this is attempted. The majority of cells remain immunoreactive with RC2 in the presence of FGF-2, but TuJ1-immunostained immature neurons, which are never apparent in FGF-2 plus EGF, can be detected at low frequency (Figures 3G–3J). Upon subsequent withdrawal of FGF-2, the neuroblast marker doublecortin is expressed by a sub-population of cells (unpublished data). Many cells of neuronal morphology that express a diagnostic neuronal immunophenotype then appear (see Results**)**, especially in presence of the neuronal viability supplement B27 [41]. These data suggest that upon release from EGF stimulation, the combination of laminin plus FGF-2 primes NS cells for neuroblast commitment with differentiation ensuing on mitogen withdrawal.

### Neuronal Differentiation of NS Cells

**Figure 4:**
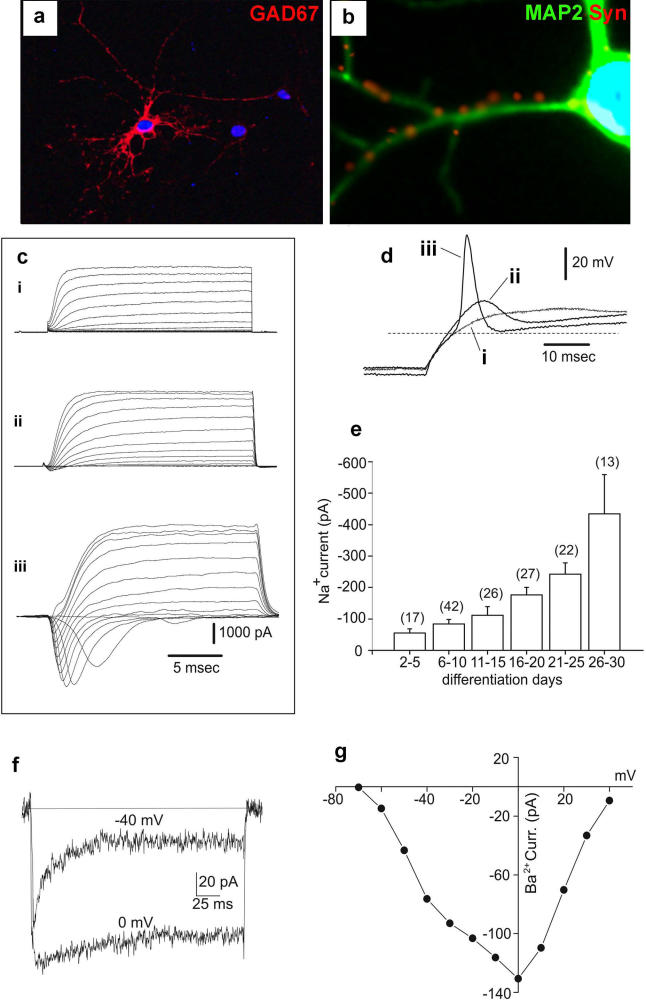
Phenotype and Electrical Activity of NS Cell–Derived Neurons (A) This LC1 NS cell–derived neuron at 27 d of differentiation displays mature morphology and expresses GAD67. (B) Expression of MAP2/synaptophysin after 7 d of differentiation. (C) Superimposed inward and outward current tracings obtained at different membrane potentials (between −70 and +40 mV from a holding potential of −90 mV), from NS cell–derived neurons after differentiation for 6 (i), 20 (ii), and 30 d (iii). (D) Superimposed voltage responses obtained following injection of depolarising rectangular current pulses in the same three cells (i–iii) by switching from voltage- to current-clamp immediately after current recordings shown in (c) were obtained. The dashed line represents a voltage level of −60 mV. (E)Average Na^+^ currents elicited at −20 mV from cells cultured in differentiating medium for increasing times as indicated by labels. Bars indicate SE. (F) Superimposed inward currents elicited at −40 mV and 0 mV in 10 mM Ba^2+^ and in the presence of TTX; the holding potential was −90 mV. (G) Current/voltage relationship from the same cell as in (F).

To assess the frequency of cells within NS cultures that are capable of neuronal differentiation, we plated NS-5 cells at clonal density on laminin, expanded for 12 d in EGF/FGF-2 followed by FGF-2 alone for 5 d, then a further 7 d in B27-supplemented media without growth factor. Every colony (126/126) produced TuJ1-positive cells (Part t in Figure 2D). These data indicate that all colony forming cells in NS cultures are competent for neuronal differentiation. Most neurons are immunopositive for GAD67 (glutamic acid decarboxylase) (Figure 4A) and gamma-aminobutyric acid (unpublished data) and by 7 d a sub-population shows expression of the mature marker synaptophysin (Figure 4B).

For unambiguous assignment of neuronal identity, we investigated the electrophysiological properties of differentiated NS cells. Figure 4C shows current recordings obtained during whole-cell voltage-clamp steps to depolarising test potentials. A sizeable outward voltage-gated current, with features of a delayed-rectifier K^+^ current, is present by 6 d of differentiation (trace i). At later stages of differentiation (20 and 30 d, traces ii and iii), the current amplitude increases only slightly. By contrast, the amplitude of the inward current increases dramatically. Figure 4D shows the voltage responses elicited in the same cells after switching from voltage-clamp to current-clamp mode. The excitability properties of the cells correlate with the magnitude of the inward voltage-gated conductance. Thus, an overshooting action potential with a relatively fast depolarisation rate was elicited in the cell differentiated for 30 d (trace iii). The fast inactivating inward current was completely blocked by the selective Na^+^ channel blocker tetrodotoxin (1 μM) and peaked at a test potential between −20 and −10 mV (unpublished data), typical features of voltage-gated Na^+^ currents in neurons. The Na^+^ current amplitude at −20 mV develops during differentiation (Figure 4E). The regenerative potential (ΔV measured between the threshold and the peak) elicited by the Na^+^ current under current clamp conditions ranged between 0 and +20 mV during the first 15 d (*n =* 6), but after 25 d reached values between +30 and +70 mV (*n =* 6). Voltage-gated Ca^2+^ channel conductances were also detected (Figure 4F). The fast activating and relatively fast inactivating (τ_h_ = 21 ms) current component elicited at −40 mV is reminiscent of the neuronal low-voltage activated (LVA) Ca^2+^ channel current [42]. By contrast, the Ba^2+^ current elicited at 0 mV, displaying a slow (τ_h_ = 73 ms) and incomplete inactivation, has the typical features of the neuronal high-voltage activated (HVA) Ca^2+^ channel current. The presence in this cell of two distinct, LVA and HVA, Ca^2+^ channel conductances is confirmed by the current-voltage relationship (Figure 4G). On average, the LVA current peaked at −40 mV, while the I/V relationship for the HVA current peaked at 0 mV. A HVA Ba^2+^ current was detectable in 19 out of 27 cells, while a LVA current component was measured in 60% of the cells already expressing a HVA Ca^2+^ current (*n =* 13). In summary, NS cell–derived neurons are electrophysiologically active, exhibiting excitability properties and underlying voltage-gated Na^+^ and Ca^2+^ conductances typical of maturing nerve cells.

### NS Cells Exhibit Phenotypic Similarities to Radial Glia

**Figure 5:**
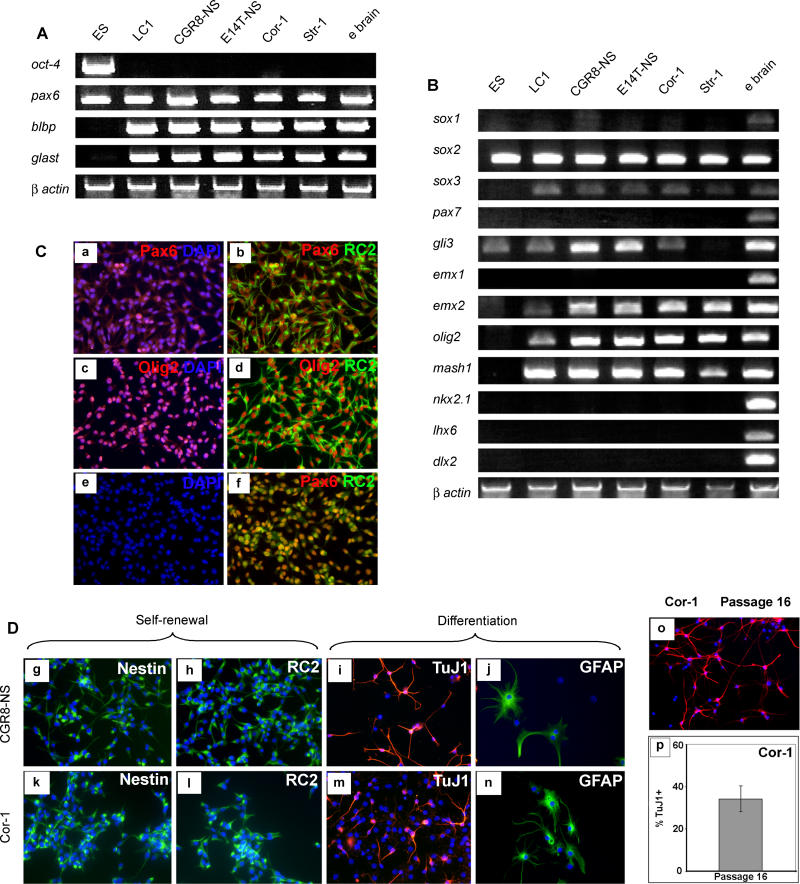
ES Cell–Derived or Forebrain-Derived NS Cells are Similar to Radial Glia NS cells were derived from independent ES cell lines (CGR8, E14Tg2a) or primary cortical (Cor-1) and striatal (Str-1) tissue. (A) RT-PCR of stem cell/radial glia markers. (B) RT-PCR for pan-neural and region-specific transcriptional regulators. (C) Double immunostaining for Pax6 and Pax6/RC2 (a,b), Olig2 and Olig2/RC2 (c,d) and Olig2/Pax6 (f). DAPI only for Olig2/Pax6 (e). (D) The ES cell–derived line (CGR8-NS) and foetal cell–derived line (Cor-1) are indistinguishable from LC1 by morphology and NS cell/radial glial marker immunoreactivity (g,h,k,l), and can each differentiate into neurons (i,m) and astrocytes (j,n). (E) The ability of Cor-1 to generate neurons (TuJ1+) is retained after 16 passages, more than 30 generations (p,o).

Undifferentiated NS cells were then examined in more detail to gauge their developmental identity. By RT-PCR and Genechip analyses, they were found to lack pluripotency marker genes such as *Oct-4,* *nanog,* and *Eras* and markers of mesoderm or endoderm (Figure 5A and unpublished data). They express Pax6, Glast, and BLBP mRNAs (Figure 5A), and are immunopositive for nestin, RC2, vimentin, 3CB2, SSEA1/Lex1, Pax6, and prominin (Figures 5C, 5D, and S1). This set of markers is considered diagnostic for neurogenic radial glia, precursors of both neurons and astrocytes during development of the nervous system [43–45]. Retinoic acid treatment of ES cells has recently been shown to induce radial glia-like cells as transient intermediates during neuronal differentiation [46,47]. Propagation of these radial glia cells was not described in these reports, however.

NS cells generally have elongated bipolar morphology, lamellate extensions, end-feet, and oval nuclei anticipated for radial glia [48]. Some more flattened cells and rounded cells with short extensions are also present. Immunostaining for the metaphase marker phosphorylated histone H3 indicates that the highly compacted cells are mitotic (unpublished data). Time-lapse videomicroscopy demonstrates a dynamic interconversion of morphology (Videos S1 and S2). In addition, time lapse reveals that NS cell nuclei undergo pronounced migration up and down the entire length of the cell process (Video S3). Such interkinetic nuclear migration is a well-characterised feature of neuroepithelial and radial glia cells in vivo [49]. It is striking to observe this occur in isolated cells, indicating that nuclear movement is a cell-autonomous property independent of cell–cell contacts or epithelial architecture.

All NS cells examined express the same panel of radial glia markers (Figures 5A and S2), plus the neural precursor markers Sox2, Sox3, and Emx2, and the bHLH (basic helix–loop helix) transcription factors Olig2 and Mash1 (Figure 5B). Although suggestive overall of telencephalic character, this set of markers does not neatly correlate with a specific regional identity. The presence of Olig2 and Mash1 is not characteristic of dorsal forebrain, and may reflect the ex vivo environment and a response to FGF-2. Indeed, *Olig2* has recently been found to be induced in spinal cord precursor cells and in neurospheres cultured in FGF-2, indicative of a relaxation of developmental specification [50,51]. Absence of Sox1, but maintenance of Sox2, is a noteworthy feature of NS cells, in view of the postulated determinative function of these transcription factors [52]. Whilst Sox1 marks all early neuroectodermal precursors, our data show that it is not retained in stem cells, where Sox2 may play the key role. NS cells also express Emx2, which is implicated in expansion of neural precursor cells [53,54] (Figures 5B and S1). Also, Dlx2, expressed in transit amplifying neuroblasts, but not stem cells in the sub-ventricular zone [55], is not detected in NS cells.

Most importantly, NS cells appear highly homogenous, staining uniformly for the various antigens examined. Double immunohistochemistry shows co-expression of Pax6/RC2, Olig2/RC2, Olig2/Pax6 (Figure 5C), and Pax6/Emx2 (Figure S1) in virtually every cell. Together with the absence of GFAP and TuJ1, this is indicative of pure symmetrical self-renewal divisions.

### Derivation of NS Cells from Foetal Brain and from Neurospheres

ES cells are adapted for in vitro self-renewal [56], and this could in turn predispose for the propagation of derivative tissue stem cells. However, multiple phenotypic characteristics suggest that NS cells may be culture analogues of neurogenic radial glia. We therefore examined whether NS cell derivation depended on an epigenetic configuration carried over from ES cells or if they could be isolated from foetal neural tissue. Primary foetal CNS cells were harvested from embryonic day (E)16.5 mouse foetal forebrain and cultured in NS-A plus EGF/FGF-2. Initially, the cells adhered poorly to plastic and spontaneously formed floating clusters. After 6–7 d, these clusters were transferred to fresh dishes where they settled onto gelatin-coated plastic. Fourteen days later, outgrowths were trypsinised and re-plated. In three separate experiments, cells morphologically identifiable as NS cells proliferated and were subsequently expanded into continuous cell lines. These foetal brain derivatives express the same radial glia and neurogenic markers as the ES cell–derived NS cells (Parts k and l in Figure 5D, and Figure S2) and show consistent mRNA profiles by RT-PCR (Figures 5A and 5B) and by GeneChip analyses (unpublished data). They are likewise competent for neuronal and astrocyte differentiation (Parts m and n in Figure 5D). Cortex-derived Cor-1 cells were plated as single cells, and then colonies were subjected to sequential growth factor withdrawal as described for NS-5. Every colony produced TuJ1-positive neurons. This indicates that all clonogenic cells in the Cor-1 culture are neurogenic. These Cor-1 cells were also readily sub-cloned and continuously expanded from individual cells with retention of phenotypic markers of radial glia, as well as neuronal and astrocyte differentiation potential (Figure S2), indicative of self-renewal. Thus, NS cells derived from foetal brain share the key properties of ES cell–derived NS cells.

It has been reported that cells expressing radial glia markers persist in neurospheres [44] and that neurospheres can “differentiate” into radial glia [57]. It has also been shown that neurospheres can be obtained, albeit inefficiently, by continuous suspension culture during neural differentiation of ES cells [58]. We reasoned that NS cells may in fact be the resident stem cells within the neurosphere. Frozen/thawed, passage 40, mouse neurospheres derived from foetal forebrain were allowed to attach to gelatin-coated plastic in the presence of EGF and FGF-2. Bipolar cells outgrew that are indistinguishable from NS cells. These cells can be serially propagated as uniformly RC2-positive, GFAP-negative populations and then induced to differentiate into astrocytes or neurons (Figure S3). We conclude that radial glia-like cells present in neurospheres give rise to NS cells in adherent culture in the presence of FGF-2 plus EGF. Conversely, we observe that NS cells of either ES cell or foetal brain origin will readily form neurospheres, if detached from the substratum either mechanically or due to overgrowth. This suggests that NS cells/radial glia cells are likely the neurosphere forming stem cells. However, in contrast to adherent cultures, in neurospheres, stem cells constitute only a fraction of the cell population. This is presumably because aggregation induces differentiation, analogous to embryoid body differentiation by ES cell aggregates [59].

### Survival and Differentiation of NS Cells Transplanted into the Rodent Brain

**Figure 6:**
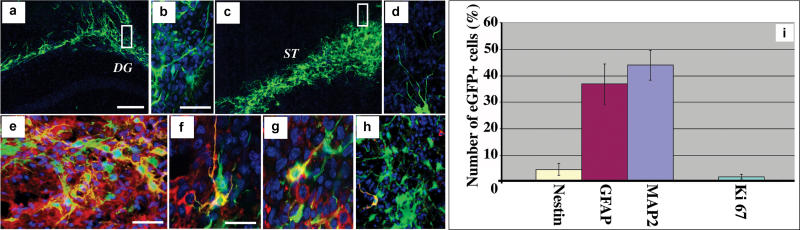
NS Cells Incorporate and Differentiate within the Adult Brain (A–H) Confocal images of LC1 NS cells, lentivirally transduced with enhanced GFP, 4 wk post-grafting into hippocampus (A,B) or striatum (C–H). (B) and (D) show higher magnification of the insets in panels (A) and (C), respectively. Examples of enhanced GFP grafted NS cells (green) showing co-expression (yellow) of the neuronal markers TuJ (E, red) or MAP-2 (F, red), astroglial marker GFAP (G, red), neural progenitor marker nestin (H, red). (I) Quantitative analysis of graft-derived neuronal (MAP2), astroglial (GFAP), progenitor (Nestin), and proliferating (Ki67) cells, 4 wk after transplantation into adult mouse striatum. Data are means (± standard deviation) of at least 500 enhanced GFP+ cells from five independent animals. DG, Dentate Gyrus; ST, Striatum. Scale bars: A,C, 100 μm; B, D, E, 40 μm.; F–H, 20 μm.

We investigated the behaviour of NS cells upon transplantation into mouse brain. ES cell–derived LC1 cells, transduced with a lentiviral enhanced GFP expression vector, were introduced into the developing brain by intra-uterine injection at E14.5 [60]. Animals were sacrificed after birth and the presence of GFP-positive cells was examined in brain sections. Mainly, GFP-labelled NS cell progeny migrated into striatum and cortex, with a few cells in the ventral telencephalon and olfactory bulbs. Immunohistochemical analyses revealed co-expression of enhanced GFP with the precursor marker nestin, neuronal markers TuJ, NeuN, and MAP2, and in lesser numbers with GFAP (Figure S4). NS cells were also injected into the adult mouse hippocampus and striatum. In this case, GFP-positive cells remained localised to the vicinity of the injection site. Four weeks after grafting, 44.4 ± 5.7% of GFP-expressing cells had neuronal morphology and were immunopositive for MAP2, 37.4 ± 6.1% expressed GFAP, and 4.2 ± 1.9% retained expression of nestin (Figure 6). A fraction of cells showed weak expression of either GFAP or neuronal markers that did not justify definitive classification but may be indicative of early stages of differentiation. The proliferative marker Ki67 was detected in only 1.0 ± 0.6% of GFP-positive cells, indicating that NS cells withdraw from the cell cycle in vivo. Well-differentiated GFP-labelled cells are also readily detected by GFP immunostaining in animals sacrificed 15 wk (*n =* 4) and 6 mo (*n =* 4) after grafting. We observed no histological evidence of unregulated proliferation or tumour formation in a total of 43 brains examined from 1–6 mo after transplantation, all with donor contributions. Furthermore, NS cells grafted to mouse kidney capsules did not proliferate or give rise to teratomas. These data indicate that NS cells can survive and differentiate in both foetal and adult brain environments and, unlike ES cells [61], they do not give rise to teratomas. Moreover, the relatively high frequency of neuronal differentiation is in contrast to grafts of passaged neurospheres, which appear predisposed in favour of glial differentiation [19,62]. The latter may reflect the incidence of committed glial progenitors present in neurospheres and seemingly absent in NS cell cultures

### Derivation of NS-Like Cells from Human ES Cells and Foetal Cortex

**Figure 7:**
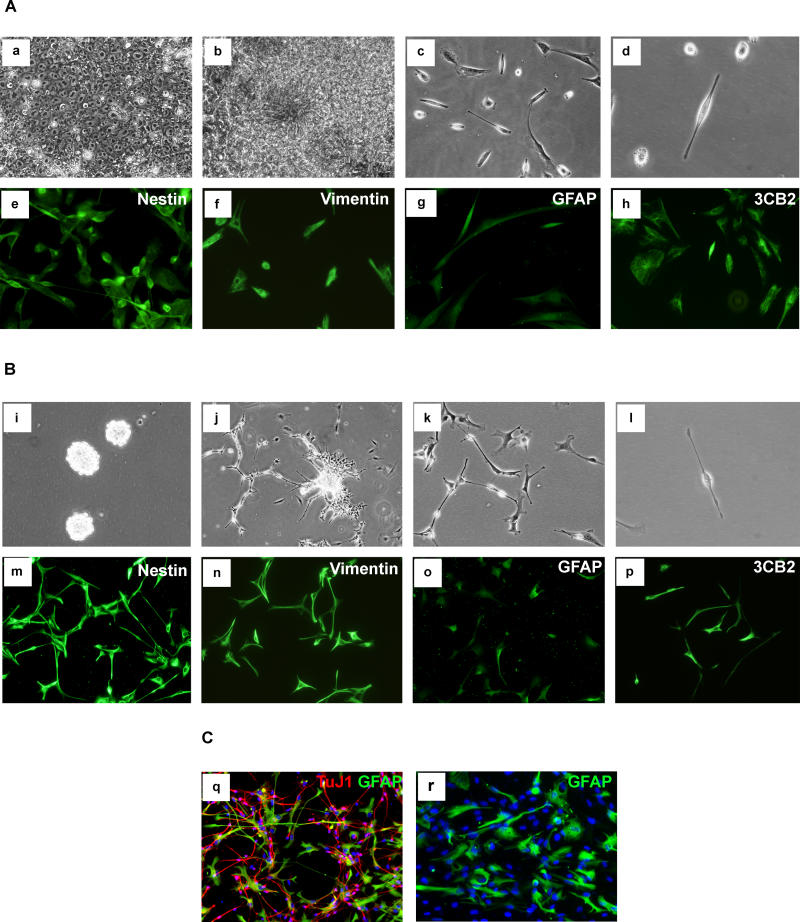
Human ES Cell or Foetal-Derived NS Cells (A) Derivation from human ES cells: human ES cell primary culture (a), differentiation of human ES cells into neural-rosette structures (b), passage 9 in NS expansion medium (c), individual cells exhibit radial glial morphology (d), and immunostaining for NS cell/radial glia markers (e–h). (B) Derivation from human foetal forebrain: floating clusters (i) generated from cortex, attachment and outgrowth (j), passage 5 in NS expansion medium (k), radial glia morphology (l), and NS cell/radial glial markers (m–p). (C) Differentiation of human foetal NS cells: TuJ1 positive neuronal cells generated by sequential growth factor withdrawal (q), and GFAP positive astrocytes induced by exposure to serum (r).

Finally, we investigated whether similar NS cells could be isolated from human sources. In the process of attempting to derive human ES cells from donated supernumerary embryos, we observed, after 5–6 wk of culture, extensive spontaneous differentiation into rosette structures typical of neuroepithelial cells (Parts a and b in Figure 7A). These colonies were manually transferred into feeder-free culture in NS expansion medium. After a further 3–4 wk, bipolar cells similar to NS cells emerged from these cultures (parts c and d in Figure 7A) and have been continuously cultured for 5 mo. We also sourced Carnegie stage 19–20 human foetal cortex from elective terminations. Following tissue dissociation, cells initially formed floating aggregates that after 7 d were re-plated and allowed to attach to gelatin-coated plastic as for derivation of NS cells from mouse foetal brain (Parts i and j in Figure 7B). Proliferating cultures were established (Part k in Figure 7B). Human cultures derived from either ES cells or foetal forebrain were characterized by the presence of flattened cells associated with the bipolar cells. However, all cells express immature precursor markers nestin, vimentin, and 3CB2 (Figures 7A and 7B). Time-lapse monitoring confirmed that the two cell morphologies are plastic and interconvertible (Video S4). These human cells exhibit moderate levels of GFAP unlike mouse NS cells, but consistent with the known activity of the human GFAP promoter in radial glia [48,63]. They proliferate more slowly than the mouse cells, with doubling times of 5–10 d. After sequential withdrawal of EGF and FGF-2, they generate mixed populations of TuJ1-positive neuron-like cells and GFAP-positive cells (Part q in Figure 7C). Near-pure populations of cells with typical astrocyte morphology and intense GFAP immunoreactivity are readily produced after exposure to serum (Part r in Figure 7C). These data suggest that NS cells may be obtained from human as for mouse, although species-specific refinements may be required for optimal propagation and differentiation.

## Discussion

The findings reported here establish that the growth factors EGF and FGF-2, plus insulin provided in N2, are sufficient to sustain robust expansion of neural stem cells in defined monoculture, liberated from any requirement for a specific cellular niche. Previous propagation of mammalian CNS precursor cells [64] has predominantly relied on short-term progenitor cell cultures [22,25], genetic immortalization of progenitors [23], or maintenance of stem cells within neurospheres [7]. Progenitor cells from the adult rat hippocampus have been propagated in adherent culture with FGF-2 whilst retaining ability to form neurons [65], albeit at low efficiency without inductive astrocytes [66]. These hippocampal-derived cells are heterogeneous by immunohistochemical staining and “undergo asymmetric cell divisions to continually replenish the supply of multipotent progenitors” [65]. In contrast, the uniformity and stability of marker expression in NS cells, combined with the long-term retention of neuronal differentiation efficiency, indicate that differentiation is completely suppressed by FGF-2 plus EGF in adherent culture. Consequently, the NS cells undergo continuous symmetrical self-renewal divisions.

The critical difference between the adherent culture system we describe and those used by others appears to be the use of EGF in addition to FGF-2. Although often applied to neurosphere cultures, EGF is typically omitted for attached cells. We find that continuous provision of EGF is essential for the derivation and propagation of NS cells, whether sourced from mouse ES cells or foetal brain. This may be related to the role of ErbB signalling in establishment of radial glia in vivo [67] and crosstalk with the Notch pathway [68]. Also, EGF functions to suppress apoptosis in NS cells (Figure 4B and 4D).

The evidence suggests that NS cells are closely related to a radial glia lineage. Whether any radial glia actually function as self-renewing stem cells in vivo is uncertain, since they exist only transiently during foetal development. However, recent fate mapping analysis [69] indicates that a subset of radial glia give rise to the sub-ventricular zone astrocytes that function as adult neural stem cells [70,71]. A possible relationship has also been proposed between hippocampal progenitors and radial glia [72]. The derivation of NS cells may reflect this intrinsic potential of radial glia to convert into stem cells. EGF has been reported to down-regulate expression of Dlx2 in transit-amplifying cells of the sub-ventricular zone and promote their conversion into neurosphere forming cells [55]. Consistent with this, we find that Dlx2 is not expressed in NS cells. Intriguingly, evidence has recently been presented that the EGF receptor undergoes asymmetric segregation in a proportion of RC2-staining neural progenitor cell divisions in vivo [28]. The retention or loss of the EGF receptor is suggested to facilitate alternative fate choices. Retention by both daughters of full responsiveness to EGF may be a central aspect of NS cell propagation. This could represent a crucial divergence from the circumstances of radial progenitor cells in vivo that underpins self-renewal in vitro.

NS cells do not express pluripotent cell-specific transcription factors Oct-4 and Nanog, but show appropriate expression of neural genes and absence of mesoderm and endoderm-specific genes. Their close relationship to a defined endogenous neural precursor cell, radial glia, adds further interest. NS cells can be cryopreserved, and may be transiently or stably transfected by electroporation or lipofection (unpublished data), or transduced with lentiviral vectors. They may be derived from previously engineered ES cells or transgenic mice or be genetically modified after derivation, opening new windows for genetic intervention into self-renewal and lineage commitment decisions in the nervous system and for investigation of neurodegenerative processes and oncogenic transformation. The potential of NS cells to generate different neuronal sub-types has yet to be determined, but their engraftment after transplantation into the adult brain suggests the potential for delivery of cell replacement and gene therapies. Whilst long-term stability and functional integration in vivo will have to be established in future studies, the preliminary data of human analogues to mouse NS cells provides encouragement for this approach.

In the context of fundamental stem cell biology, homogenous expansion of any stem cell in defined conditions has hitherto been the exclusive preserve of the ES cell. Ex vivo propagation of tissue stem cells has invariably been accompanied by differentiation, often interpreted as reflecting an intrinsic bias towards asymmetric division. The findings reported here show that this is definitively not the case for at least one class of neural stem cell. NS cells undergo sustained symmetrical self-renewal divisions with complete suppression of differentiation in response to FGF-2 and EGF. They thus provide a directly accessible system for molecular characterization and experimental manipulation of the stem cell state. Therefore, NS cells offer the first known tissue stem cell resource for direct comparison with ES cells in order to delineate common and distinct mechanistic features of lineage-restricted and pluripotent stem cells.

## Materials and Methods

### Mouse cell culture and differentiation

ES cells and neural differentiation are detailed elsewhere [38]. The LC1 and other ES cell–derived NS cells were routinely generated by re-plating d 7 adherent neural differentiation cultures (typically 2–3 × 10^6^ cells into a T75 flask) on uncoated plastic in NS-A medium (Euroclone, Milan, Italy) supplemented with modified N2 [36] and 10 ng/ml of both EGF and FGF-2 (NS expansion medium). Over 3–5 d, cells formed aggregates that, after harvesting and sedimentation to remove debris, subsequently attached to fresh plastic and outgrew NS cells. After addition of 0.5 μg/ml of puromycin to differentiating adherent cultures at d 7, 46C-NS cells were generated. Cells were re-plated 3 d later into an uncoated T75 flask in N2B27 media with 10 ng/ml of both EGF and FGF-2 (Peprotech, Rocky Hill, New Jersey, United States) in the absence of puromycin. To derive clonal lines, including NS-5, single cells were plated into 96-well microwell plates (Nalge Nunc International, Rochester, New York, United States) by limiting dilution. and the presence of one cell per well was scored 1 h after plating.

For derivation directly from foetal CNS, primary cultures were generated using standard protocols from cortex or striatum of E16.5 mouse embryos and subsequently allowed to attach on flasks treated with 0.1% gelatin. Outgrowing cells were then expanded on gelatin using NS expansion medium. Clonal derivatives of the cortical line Cor-1 were established by plating at very low density (1,000 cells per 9-cm plate) and expanding individual colonies.

For derivation from established neurospheres, derived from E14 foetal brain and maintained for 40 passages in EGF plus FGF-2, cultures were dissociated to single cells using Accutase (Sigma, St. Louis, Missouri, United States) and plated at 10^4^ cells/ml on gelatin-coated culture flasks in NS expansion medium.

For passaging established NS cell lines, we routinely used trypsin/EDTA or PBS and split cells 1:3 to 1:5 every 2–3 d. For astrocyte differentiations, NS cells were re-plated onto 4-well plates at 1 × 10^5^ cells/well in NS-A medium supplemented with 1% fetal calf serum or 10 ng/ml BMP4 (R&D Systems, Minneapolis, Minnesota, United States). For neuronal differentiation, 5 × 10^4^ NS cells were plated into poly-ornithine/laminin treated wells in NS-A supplemented with FGF-2 alone. After 7 d, the media was switched to NS-A supplemented with B27 (GIBCO, San Diego, California, United States) without growth factor. Half of the medium was exchanged every 2–3 d during the differentiation. For clonal differentiation, 1,000 cells from NS-5 or Cor-1, cultures were plated in 10-cm plates pre-treated with laminin, expanded for 12 d in EGF/FGF-2, and differentiated in situ as above. For electrophysiological studies, 1.5 × 10^5^ NS cells were plated into poly-L-ornithine-treated 35-mm dishes in NS-A medium supplemented with N2 and B27 (both at 0.5%) and FGF-2 (5 ng/ml). After 7 d, the medium was switched to the mix NS-A:Neurobasal (1:1), supplemented with B27 (GIBCO) without growth factors. To sustain neuronal maturation, after a further 7 d, the medium was switched to the mix NS-A:Neurobasal (1:3) supplemented with B27 (GIBCO) and brain derived neurotrophic factor (20 ng/ml) and nerve growth factor (R&D Systems; 50 ng/ml). Throughout neuronal differentiation, half of the medium was replaced every 2–3 d. Further details of NS cell derivation, propagation, and differentiation are provided in Protocol S1.

### Characterisation of NS cells

Immunocytochemistry was performed using appropriate TRITC or FITC secondary conjugates and nuclear counterstaining with DAPI. Primary antibodies were used at the following dilutions: Nestin (1:10), Vimentin (1:50), Pax6 (1:5), 3CB2 (1:20), RC2 (1:50) (DSHB, Iowa City, Iowa, United States); TuJ (1:200) (Covance, Berkeley, California, United States); GFAP (1:300) (poly and mono, Sigma); MAP2 (1:200) (Chemicon, Temecula, California, United States; and Becton Dickinson, Palo Alto, California, United States); NeuN (1:200), gamma-aminobutyric acid (1:200), Gad65/67 (1:200) (Chemicon); Synaptophysin (1:200) (Sigma); Doublecortin (1:200) (Santa Cruz Biotechnology, Santa Cruz, California, United States), caspase-3 active (1:300) (R&D Systems), Olig2 (1:5000) (H. Takebayashi); Emx2 (1:2000) (A. Corte); BLBP (1:500) (N. Heins); prominin/mAb13A4 (1:200) (W. Huttner). Negative controls were ES cells, differentiated NS cells, or secondary alone. For RT-PCR, total RNA was extracted using RNeasy kit (Qiagen, Valencia, California, United States), and cDNA was generated using Superscript II (Invitrogen, Carlsbad, California, United States). PCR was performed for 30 cycles for all markers except β-actin (25 cycles). Details of primers and amplicon size are provided in Table S1. For metaphase spreads, cells were treated with 5 ml of 0.56% KCl for 20 min, fixed in methanol:acetic acid (3:1) on ice for 15 min, spread onto glass slides, and stained with TOPRO-3 (Molecular Probes, Eugene, Oregon, United States).

### Solutions for electrophysiological and patch clamp recording

Recordings were made from LC1 cells differentiated between passages 20–25. Seals between electrodes and cells were established in a bath solution consisting of (in mmoles/l): 155 NaCl, 1.0 CaCl_2_, 1 MgCl_2_, 3.0 KCl, 10 glucose, and 10 HEPES/NaOH (pH 7.4). After establishing the whole-cell configuration, for current-clamp recording and for total current recording in voltage-clamp, the pipette filling solution contained (in mmoles/l): 128 KCl, 10 NaCl, 11 EGTA, 4 Mg-ATP, and 10 HEPES/KOH (pH 7.4). For the study of voltage-gated Na^+^ channels under voltage clamp conditions, the patch pipette was filled with (in mmoles/l): 130 CsCl, 10 NaCl, 20 TEA-Cl, 10 EGTA, 2 MgCl_2_, 4 Mg-ATP, and 10 HEPES/CsOH (pH 7.4), and the extracellular solution contained (in mmoles/l): 130 NaCl, 2 CaCl_2_, 2 MgCl_2_, 10 glucose, 5 tetrethylammonium-Cl, CdCl_2_ 0.2, and 10 HEPES/NaOH (pH 7.4). For the study of voltage-gated Ca^2+^ channels, the patch pipette was filled with (in mmoles/l): 120 CsCl, 20 TEA-Cl, 10 EGTA, 2 MgCl_2_, 4 Mg-ATP, and 10 HEPES/CsOH (pH 7.4), and the extracellular solution contained (in mmoles/l): 130 NaCl, 10 BaCl_2_, 10 glucose, 5 tetrethylammonium-Cl, 10 4-AP 1, TTX 10^−3^, and HEPES/NaOH (pH 7.4). Ionic currents were recorded under voltage-clamp conditions using the patch-clamp whole-cell configuration at room temperature (20–24 °C) with an Axopatch 200B patch-clamp amplifier (Axon Instruments, Burlingame, California, United States) and digitized at sampling intervals of 26–100 μsec using a Digidata 1322A A/D converter (Axon Instruments) interfaced with an IBM-compatible PC. Stimulation, acquisition, and data analysis were carried out using the software packages: pClamp 9 (Axon Instruments) and ORIGIN 6 (Microcal Software, Northampton, Massachusetts, United States). For voltage-clamp experiments, linear components of leak and capacitive currents were first reduced by analogue circuitry and then almost completely cancelled with the P/N method. Patch pipettes were made from borosilicate glass tubing and fire polished. Pipettes had a final resistance of 3–4 MΩ when filled with internal solution. Currents were filtered at 5 KHz.

### Human embryo and foetal cultures

Research on human tissue with informed consent was approved by the Research Ethics Committee of Lothian Health Board. Frozen supernumerary human embryos were donated for research under licence R0132 issued by the Human Fertilisation and Embryology Authority. Inner cell masses were isolated by immunosurgery and cultured on human foreskin fibroblasts in medium supplemented with 15% serum replacement (Invitrogen) plus human leukaemia inhibitory factor and FGF-2 [73]. After three to four passages, cells with ES cell morphology differentiated into rosettes of neuroepithelial-like cells. These colonies were passaged into NS expansion medium without feeders or serum replacement. Human foetal tissue was obtained following elective termination with consent for research according to the Polkinghorne guidelines [74]. Cortex was dissected from Carnegie stage 19–20 foetuses and processed as described for mouse foetal tissue. In some cases, LIF (100 U/ml) was added to the expansion medium [75].

To induce neuronal differentiation, a similar protocol was followed as for mouse NS cells but with addition of brain derived neurotrophic factor (R&D Systems; 10 ng/ml) after the first 7 d without EGF, and retention of FGF-2 (5 ng/ml) until 14 d.

### Transplants

To provide a convenient reporter, LC1 cells at passage 12 were transduced with lentiviral GFP. Transplants were performed after expansion of these cells for a further 9–28 passages. Foetal surgery was performed as described previously [60]. Using a glass capillary, 5 × 10^4^ cells in a volume of 1 μl of HBSS were injected into the telencephalic vesicles of E14.5 Sprague-Dawley rat foetuses exposed under transillumination. Injected foetuses were replaced into the abdominal cavity for development to term. After delivery, animals were sacrificed at 7 d (postnatal day [P] 1, *n =* 16) and 5 wk (P30, *n =* 8) post-transplantation. For adult transplantations, 129 or CD1 mice were placed in a Kopf stereotaxic frame and received an injection of 2 × 10^5^ NS cells suspended in 5 μl of HBSS into the striatum (*n =* 22) or hippocampus (*n =* 21). Transplanted mice were sacrificed after 2 (*n =* 16) and 4 wk (*n =* 10) and perfused transcardially with 4% paraformaldehyde. Cryosections (16 μm) were stained with the following antibodies: (mouse), NeuN (1:100) and Ki67 (1:10) (Chemicon), MAP2 (1:200; Becton Dickinson), Nestin (1:5; Ron McKay); (rabbit), βIII tubulin (1:500; Covance); GFAP (1:200; Dako, Glostrup, Denmark); secondary antibodies, Texas Red (Vector Laboratories, Burlingame, California, United States) (Jackson ImmunoResearch, West Grove, Pennsylvania, United States) and AlexaFluor 488 (Molecular Probes). Sections were preserved in antifading solution and analysed on Nikon TE2000-S ECLIPSE (Nikon, Tokyo, Japan) and Bio-Rad Radiance 2100 (Bio-Rad, Hercules, California, United States) confocal microscopes. Further cohorts of animals were sacrificed after 15 wk (*n =* 4) and 6 mo (*n =* 4) and analysed by antibody staining for GFP because the fluorescence signal was very low.

## Supporting Information

Figure S1 Immunohistochemical Staining of NS Cells (A) Radial glia/neural precursor markers: GLAST (Slc1a3) (a), prominin (b), and LeX/SSEA-1 (c), in NS-5 cells (equivalent results with LC1 not shown).
(B) Co-expression of transcription factors Emx2 and Pax6: DAPI (d), Emx2 (e), and Pax6 (f) double immunostaining (g, overlay) of LC1 NS cells (equivalent results with NS-5 not shown).
(292 KB PDF).

Figure S2 Mouse ES Cell–Derived and Foetal Cortex–Derived NS Cells Uniformly Express Radial Glia Markers NS cells derived from CGR8 ES cells or from E16 foetal cortex (Cor-1 and clonal derivative Cor-1.3) were analysed for expression of the indicated markers by immunochemistry. Examination at high power shows that the radial glia markers are each expressed in almost all cells whilst they are uniformly negative for GFAP.
(641 KB PDF).

Figure S3 NS Cells Can Be Derived from Expanded Foetal Forebrain Neurospheres NS line derived from a long-term foetal neurosphere culture (40 passages) exhibits identical morphology (a) to ES-derived NS lines, expresses neural precursor cell/radial glial marker immunoreactivity (b–d), and can differentiate into neurons (e) and astrocytes (f).
(284 KB PDF).

Figure S4 NS Cells Migrate and Differentiate after Transplantation in Foetal Rat Brain Confocal image of NS cells, lentivirally transduced with enhanced GFP, 1 wk after transplantation into the ventricle of E14.5 rats (a). Donor cells migrate from the ventricle into the parenchyma in clusters and as single cells. Grafted cells show co-localization (yellow) of enhanced GFP (green) and; the neuronal marker MAP2 (b, red); astroglia marker GFAP (c, red) or; progenitor cell marker, nestin (d, red). Quantitative analysis (e) of graft-derived neuronal (NeuN and TuJ-1), astroglial (GFAP), progenitor (Nestin), and proliferating (Ki67) cells, one week after transplantation. Data are means (± standard deviation) of at least 500 enhanced GFP+ cells from five independent animals. LV, lateral ventricle. Scale bars: (a) 200 μm; (b–d), 20 μm.
(154 KB PDF).

Protocol S1 Derivation and Manipulation of NS Cell Lines (59 KB DOC).

Table S1 Primers Used for RT-PCR (74 KB DOC).

Video S1 Mouse ES Cell–Derived (E14T-NS) NS Cells Show Dynamic Morphology (Low Magnification) 3 frames/second; 22 s running time.
(7.5 MB AVI).

Video S2 Mouse ES Cell–Derived (E14T-NS) NS Cells Show Dynamic Morphology (Higher Magnification) 3 frames/second; 72 s running time.
(4 MB MOV).

Video S3 Mouse Foetal Cortex–Derived NS Cell Exhibits Interkinetic Nuclear Migration 5 frames/second; 43 s running time.
(1.3 MB AVI).

Video S4 Human Foetal Cortex–Derived NS Cells Show Dynamic Morphology, Alternating between Bipolar and Spread-Out States 10 frames/second; 20 s running time.
(2.1 MB AVI).

### Accession Numbers

The Swiss-Prot (http://www.ebi.ac.uk/swissprot) accession numbers for the genes and gene products discussed in this paper are *Ascl1* (Mash1) (Q02067), *Dlx2* (P40764), *Egf* (P01132), *Emx2* (Q04744), *Fabp7* (BLBP, B-FABP), (P51880), *Fgf2* (bFGF) (P15655), *Gad1* (Gad67) (P48318), *Gfap* (P03995), *Nanog* (Q7TN85), Nestin (Q6P5H2), *Olig2* (Q9EQW6), *Pax6* (P63015), *Pax7* (P47239), *Pou5f1* (Oct4, Oct3/4) (P20263), *Slc1a3* (Glast) (P56564), *Sox1* (P53783), *Sox2* (P48432), *Sox3* (P53784), and *Vim* (P20152).

## Abbreviations

BLBP: brain lipid binding protein
CNS: central nervous system
E: embryonic day
EGF: epidermal growth factor
ES: embryonic stem
FGF-2: fibroblast growth factor 2
GFAP: glial fibrillary acidic protein
GFP: green fluorescent protein
HVA: high-voltage activated
LVA: low-voltage activated
MAP2: microtubule associated protein-2
NS: neural stem
P: postnatal day
