# Supplementary material for: Niche-Independent Symmetrical Self-Renewal of a Mammalian Tissue Stem Cell
Source: PLoS Biol. 2005 Aug 16;3(9):e283. doi: 10.1371/journal.pbio.0030283 (PMC1184591; doi:10.1371/journal.pbio.0030283)
Supplement: Supplementary file 1 [file pbio.0030283.sg001.pdf]

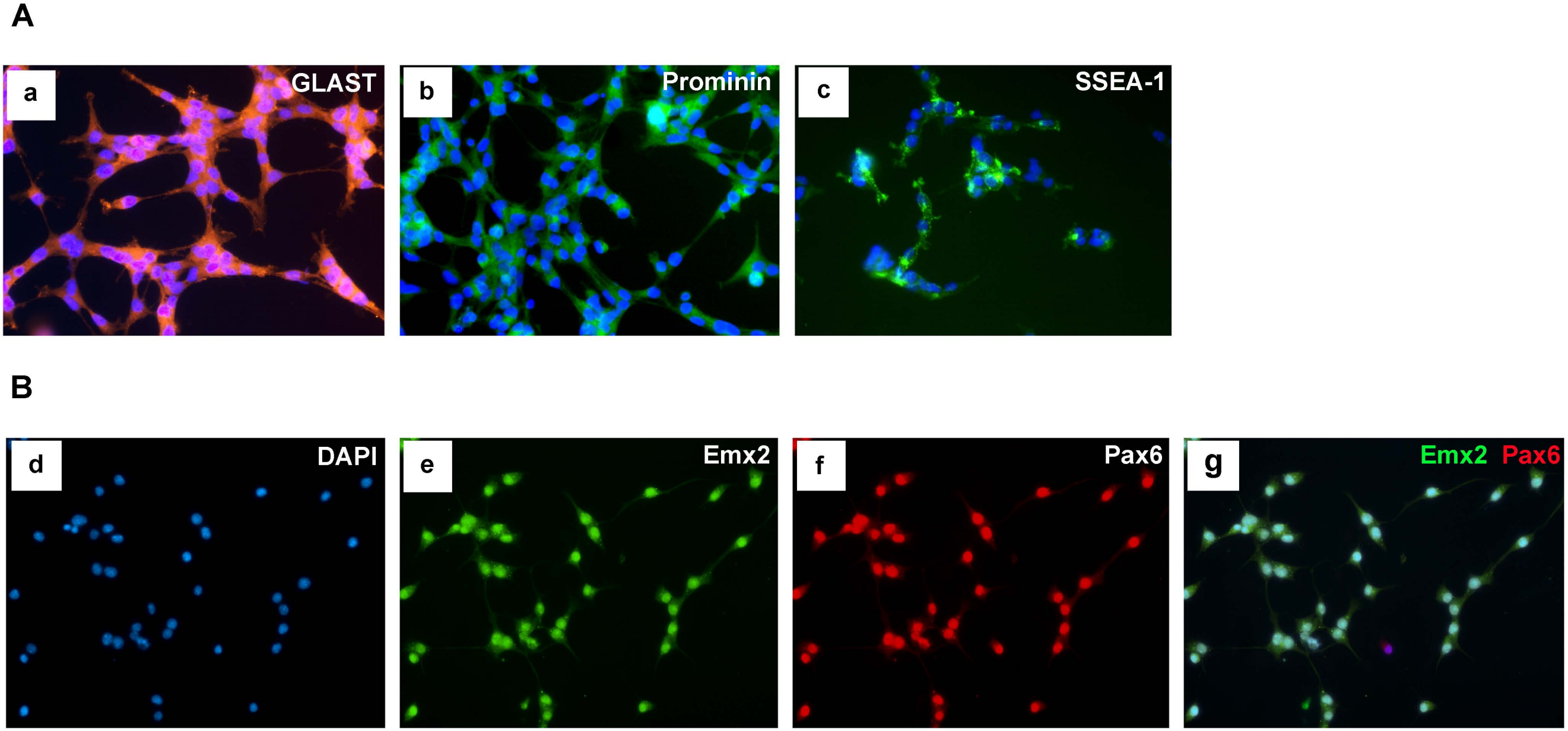
A
a
GLAST
b
Prominin
c
SSEA-1
B
d
DAPI
e
Emx2
f
Pax6
g
Emx2 Pax6
