# Supplementary material for: Niche-Independent Symmetrical Self-Renewal of a Mammalian Tissue Stem Cell
Source: PLoS Biol. 2005 Aug 16;3(9):e283. doi: 10.1371/journal.pbio.0030283 (PMC1184591; doi:10.1371/journal.pbio.0030283)
Supplement: Supplementary file 2 [file pbio.0030283.sg002.pdf]

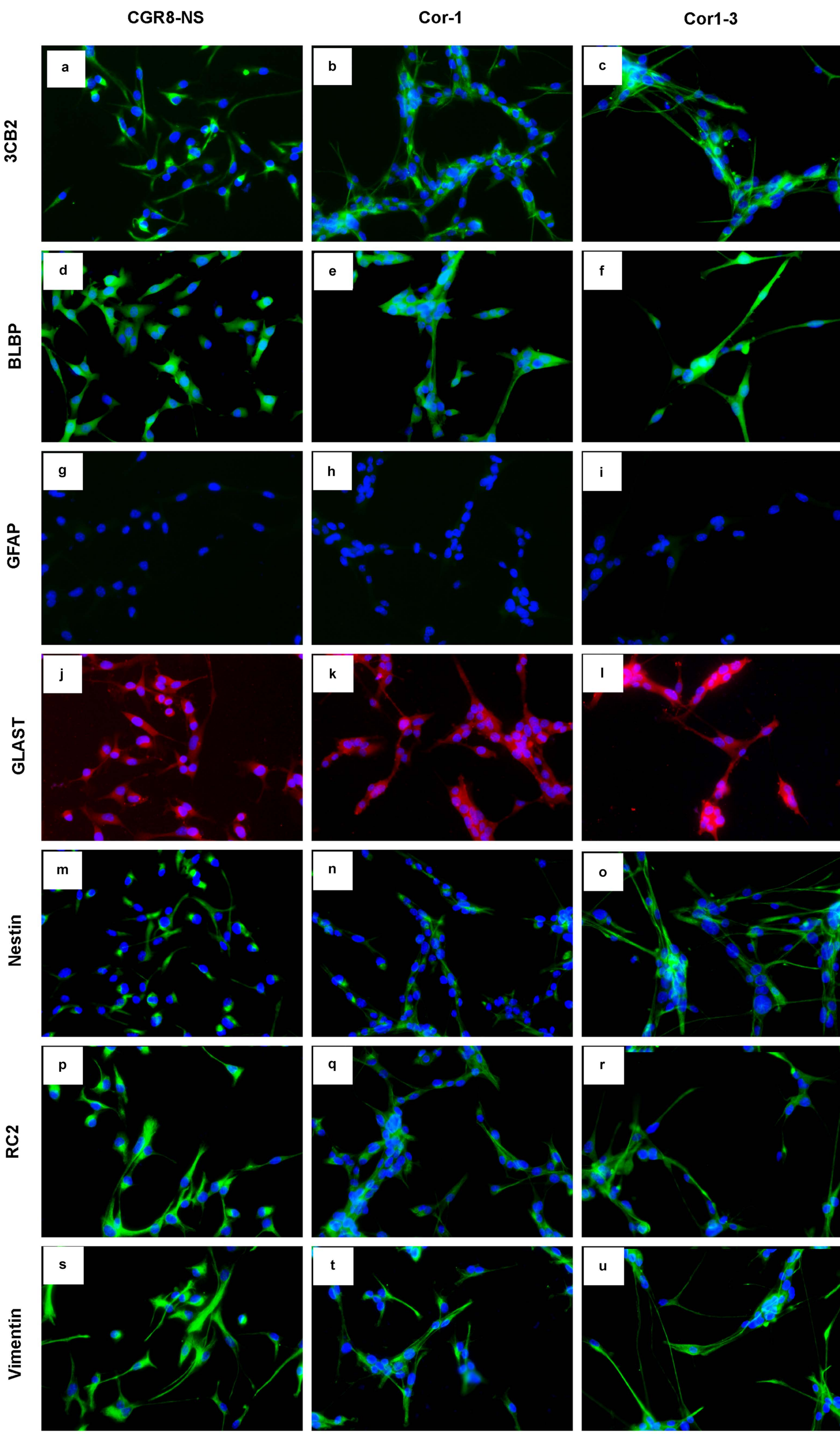

CGR8-NS
Cor-1
Cor1-3
3CB2
BLBP
GFAP
GLAST
Nestin
RC2
Vimentin
a
b
c
d
e
f
g
h
i
j
k
l
m
n
o
p
q
r
s
t
u
