# Supplementary material for: Niche-Independent Symmetrical Self-Renewal of a Mammalian Tissue Stem Cell
Source: PLoS Biol. 2005 Aug 16;3(9):e283. doi: 10.1371/journal.pbio.0030283 (PMC1184591; doi:10.1371/journal.pbio.0030283)
Supplement: Supplementary file 3 [file pbio.0030283.sg003.pdf]

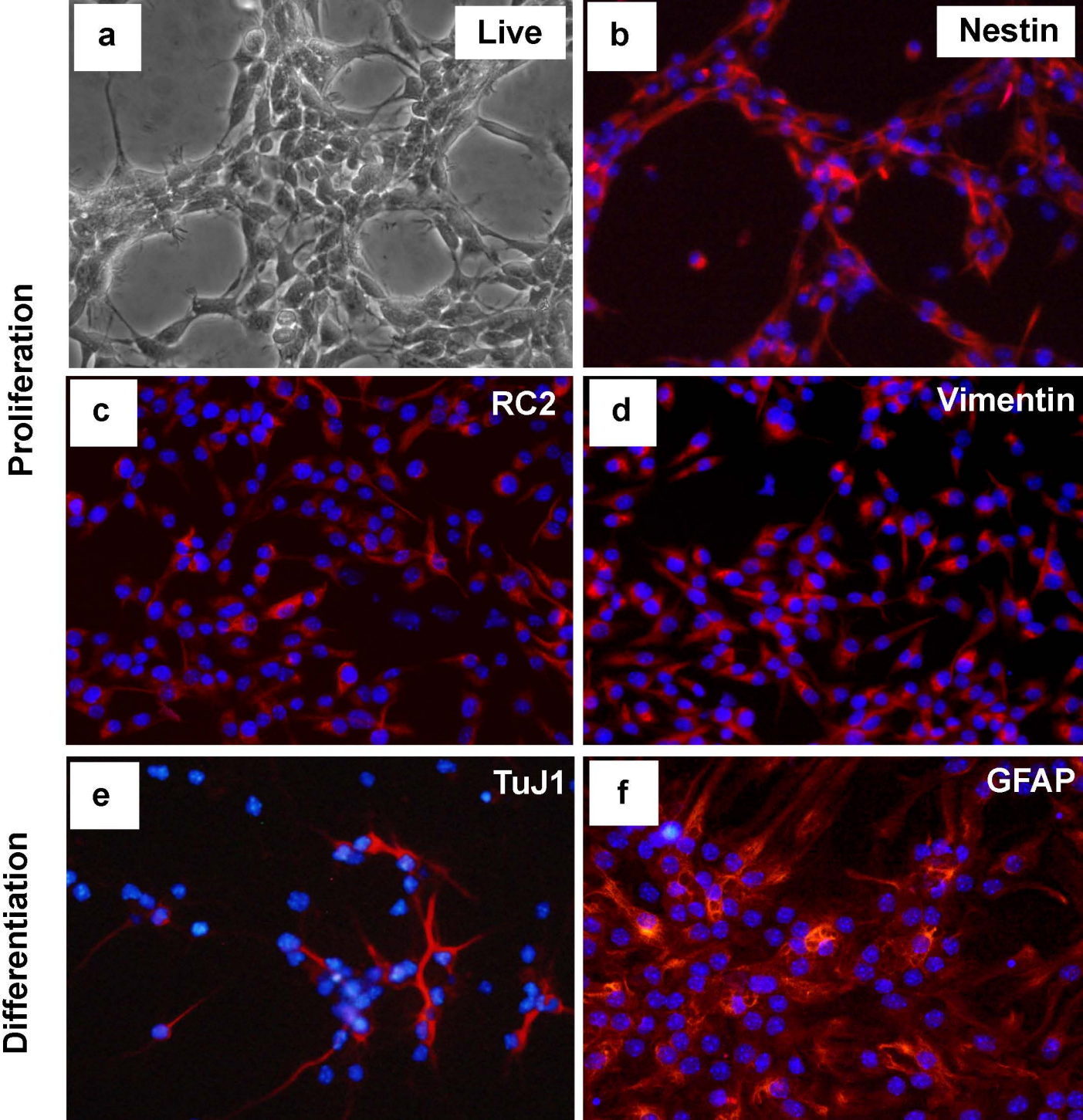

a
Live
b
Nestin
c
RC2
d
Vimentin
e
TuJ1
f
GFAP
Proliferation
Differentiation
