# Supplementary material for: Niche-Independent Symmetrical Self-Renewal of a Mammalian Tissue Stem Cell
Source: PLoS Biol. 2005 Aug 16;3(9):e283. doi: 10.1371/journal.pbio.0030283 (PMC1184591; doi:10.1371/journal.pbio.0030283)
Supplement: Supplementary file 4 [file pbio.0030283.sg004.pdf]

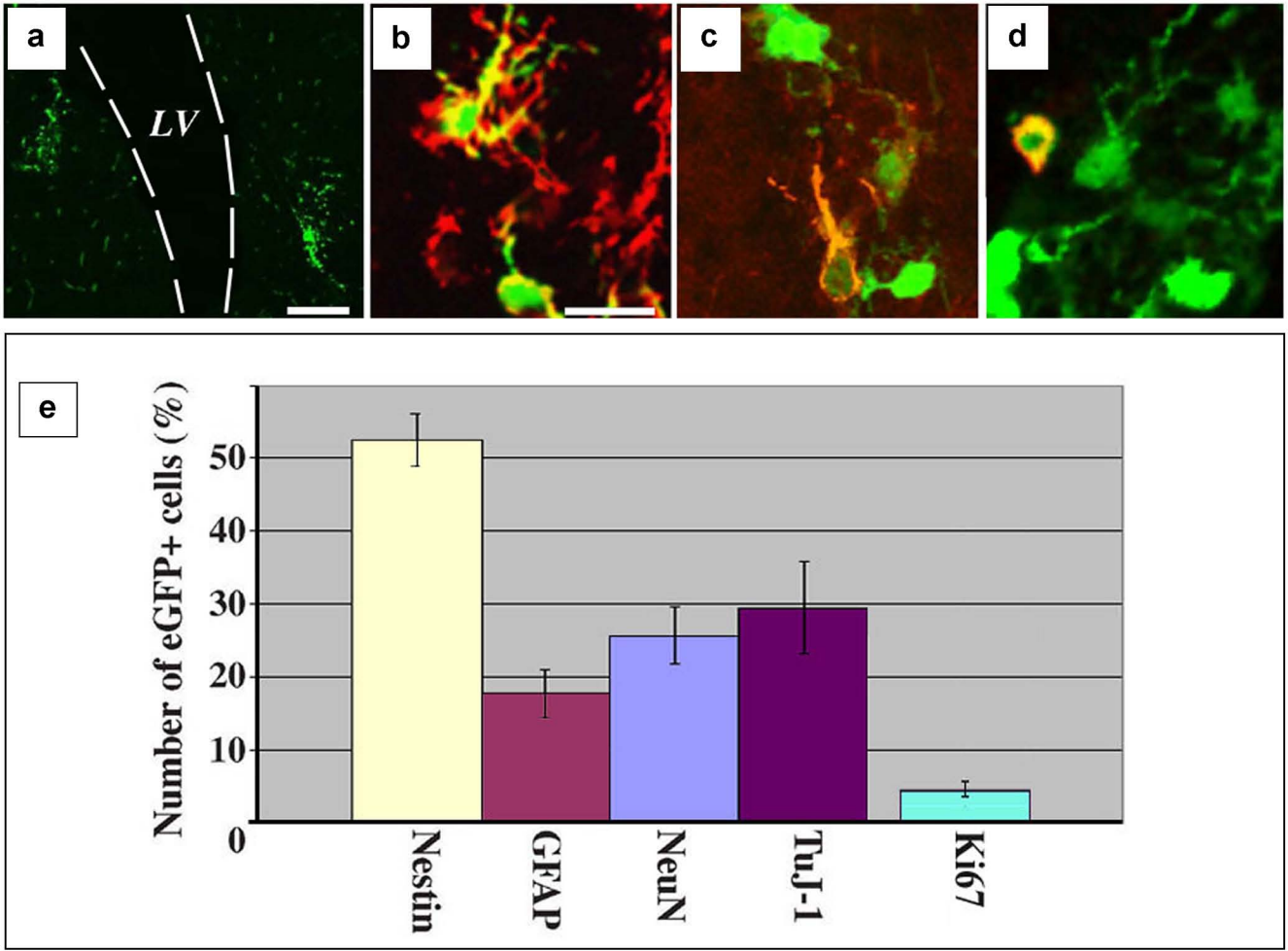
a
LV
b
c
d
e
Number of eGFP+ cells (%)
0
10
20
30
40
50
Nestin
GFAP
NeuN
TuJ-1
Ki67
