# Supplementary material for: Niche-Independent Symmetrical Self-Renewal of a Mammalian Tissue Stem Cell
Source: PLoS Biol. 2005 Aug 16;3(9):e283. doi: 10.1371/journal.pbio.0030283 (PMC1184591; doi:10.1371/journal.pbio.0030283)
Supplement: Supplementary file 5 [file pbio.0030283.sd001.doc]

**Protocol S1	Derivation and manipulation of NS cell lines**

**(Conti, L., Pollard S.M., et al.)**

**Derivation of mouse NS cell lines from ES cells**

ES cells can efficiently be converted to Sox1 expressing neural progenitors in adherent monolayer culture [1]. Detailed protocols and troubleshooting and for this ES cell differentiation are described elsewhere [2]. Briefly, ES cells are routinely cultured under feeder-free conditions in medium supplemented with 10% foetal calf serum and 100 U/ml recombinant leukaemia inhibitory factor (LIF) on gelatin-coated tissue culture plastic [3]. Undifferentiated ES cells are expanded to ~80% confluence in a T75 flask (Iwaki), trypsinised and resuspended in N2B27 media [2]. Cells are plated onto 9cm plates (Iwaki) that have been coated with a 0.1% gelatin solution (Sigma) for at least 10 mins then allowed to dry. As initial plating density is a crucial parameter for efficient neural induction, and can vary between ES cell lines, we routinely seed several different cell densities (0.8 x 10^6^, 1 x 10^6^ and 1.2 x 10^6^) per plaste. Culture medium is changed each day, in the process removing detached or dead cells. Under these conditions 50-80% of cells will undergo neural lineage specification within 4-5 days, and with overt neuronal differentiation detectable from day 5 onwards.

Conversion of heterogeneous progenitor cultures to homogeneous NS cell lines can be achieved as follows. Day 7 differentiated cultures are trypsinised and 2-3 x 10^6^ cells are re-plated into an uncoated T75 flask in NS expansion media, comprising NS-A media (Euroclone) supplemented with L-glutamine, 2mM final (Gibco), modified N2 supplement (freshly prepared in house) [2] and 10ng/ml of both mouse EGF (Peprotech) and human FGF-2 (Peprotech). Expansion media can be stored at 4^o^C for up to 4 weeks. Within 2-3 days the flask will contain many thousands of floating aggregates in suspension culture (absolute number varies according to efficiency of initial ES cell differentiation). Cell aggregates are harvested by mild centrifugation or allowed to settle under gravity in a 30ml universal tube for 10mins. This step removes debris and dead cells, thus enriching for NS cell founders, and ensures complete media exchange. Cells are replated in 10ml of fresh NS expansion medium onto a gelatin coated T75 flask (Iwaki). After a further 3-7 days, cell aggregates will attach to the flask and shortly thereafter cells outgrow with characteristic bipolar NS cell morphology (see manuscript Figure 1). Following extensive outgrowth of cells (a further 3-4 days) the entire population is trypsinised and re-plated as single cells onto a gelatin coated T75 flask in expansion medium. Cells grow very rapidly (doubling time ~25hrs) and remain adherent. Within several passages residual differentiated and blast cells are eliminated (monitored by GFAP and TuJ1 immunostaining) and cultures are uniformly positive for NS cell markers (as described in Results section).

For 46C ES cells (*Sox1*-GFP-IRES-pac knock-in) [4] a transient selection with puromycin (0.5μg/ml) can be used to eliminate non-neural cells and derive NS cells via continuous adherent culture. 46C ES cells (1 x 10^6^ ) are plated in N2B27 media onto a gelatin coated 9cm dish to induce neural commitment. Six days later puromycin is added for 48hrs. The enriched Sox1 expressing cell population (~ 3-5 x 10^6^) is then re-plated in N2B27 medium containing EGF (10ng/ml) and FGF-2 (10ng/ml) on gelatin coated 9cm plates. The initially morphologically heterogenous Sox1 expressing population progressively acquires a Sox1 negative character and after 3-4 passages uniform NS cell morphology and marker expression.

**Derivation of mouse NS cell lines from foetal CNS and neurospheres**

Cell clusters form in suspension upon dissociation of foetal E16.5 cortex and primary culture in NS cell expansion media. These primary aggregates can be readily converted into adherent NS cell lines by plating onto gelatin-coated substrate in expansion medium. In order to promote attachment, it is important that debris/dead cells are first effectively removed by sedimentation and medium is exchanged completely. Cell aggregates will attach and outgrow over 2-5 days. Outgrowing cells can subsequently be trypsinised to single cells, re-plated and propagated in NS cell expansion medium. From passaged foetal neurospheres [5], NS cells can conveniently be established by dissociation and plating directly on gelatin coated plastic in NS expansion medium. During the initial few passages the derived NS lines have a tendency to aggregate, detach from the flask and re-form neurospheres, particularly if the cell density becomes high. Cultures should therefore be passaged at or below 50% confluence. The tendency to spontaneously aggregate is variable, but is generally reduced upon further passaging, or through establishment of clonal cell lines.

**Passage and expansion of NS cells**

Once established, NS cells are propagated in NS expansion media. NS cells are grown on gelatin coated plates/flasks and are routinely split 1 in 2 to 1 in 5. NS cells have a doubling time of around 25hrs. Cells are passaged using trypsin/EDTA or through incubation with calcium magnesium-free PBS (Sigma). For establishment of clonal lines, single cells can be deposited in gelatin-coated microwells in expansion medium. Less rigorously, cells can be plated at very low density, 1000 cells per 9cm dish. Colonies appear within two weeks and can be picked and expanded.

**Cryopreservation and recovery of NS cells**

NS cells are readily recovered following freezing/thawing. Routinely we trypsinise a 60-90% confluent T75 flask, and resuspend the pellet into 1.5ml NS expansion media plus 10% DMSO. This is then split into 3x 1ml cryotubes (Nunc) and stored at -80^o^C. NS cells are recoverable following more than 6 months storage in these conditions. For long term storage frozen vials are transferred to liquid nitrogen. NS cells are thawed by rapidly bringing the vial to 37^o^C followed by transfer to 10ml of pre-warmed NS expansion media. Cells are pelleted and then resuspended in fresh expansion media to remove DMSO. Cell recovery following cryopreservation is >95% for NS cells.

**Astrocyte and neuronal differentiation of NS cells**

Rapid differentiation of NS cells to GFAP positive astrocytes occurs within 2 days of exposure of NS cells to BMP4 (10ng/ml) or 1% FCS in NS-A (with N2, without EGF/FGF) on gelatine coated flasks/plates. Cell density is not a crucial parameter for astrocyte differentiation.

For neuronal differentiation NS cells are harvested using Accutase (Sigma) or calcium/magnesium-free PBS to detach cells and 0.5-1.0 x 10^4^ cells are re-plated into each well of a poly-ornithine/laminin coated 4-well multidish plate (Nunc) in NS-A medium supplemented with FGF-2 (5ng/ml), modified N2, and B27 (Gibco). We find that the NS-A basal medium is more permissive for neuronal differentiation, than other basal media. A half volume of medium is replaced every 2-3 days to maintain conditioning of medium. After 7 days in these conditions we exchange media to NS-A mixed with Neurobasal medium (Gibco) in a ratio of 1:1 supplemented with 0.25x N2 plus B27 and without EGF or FGF. This formulation promotes further neuronal differentiation and maturation. For longer term cultures of neurons (beyond 14 days) we exchange media to Neurobasal supplemented with B27 without N2 in the presence of BDNF (10ng/ml).

# Culture and neuronal differentiation of human NS cells

Human NS cells are expanded on 0.1% gelatine (Sigma) coated flasks/plates in expansion media as for mouse NS cells additionally supplemented with 100U/ml recombinant human leukaemia inhibitory factor (LIF). Cells have a doubling time of approximately 1 week. They are passaged with trypsin once reaching ~30% confluence and split 1 in 2. Overgrowth should be avoided to maintain monolayer culture and prevent aggregation and detachment.

For neuronal differentiation, human NS cells are harvested using Accutase (Sigma) and around 1x10^4^ cells re-plated into each well of poly-ornithine /laminin (Sigma) coated 12- well plate (Iwaki) in expansion media. Cells are expanded until they reach around 80% confluent. Neuronal differentiation is induced by removing EGF and LIF from expansion media. After 7 days in the absence of EGF and LIF, exchange media to NS-A mixed with Neurobasal media in a ratio of 1:1 (Gibco) supplemented with 0.5X N2, B27, FGF2 (5ng/ml), and BDNF (10ng/ml). After a further 7 days in these conditions, media is switched to Neurobasal media supplemented with B27 and BDNF (10ng/ml) without N2 or FGF-2. Half the medium is exchanged every 2 or 3 days as for mouse NS cells throughout this protocol. After a further 10 days cells of neuronal morphology immunoreactive with TuJ1 and MAP2 represent up to 40% of total cell numbers. Significant numbers of astrocytes are also generated, in contrast to the mouse NS cell protocol for neuronal differentiation in which few GFAP positive cells emerge.

**References**

1. Ying Q-L, Stavridis M, Griffiths D, Li M, Smith A (2003) Conversion of embryonic stem cells to neuroectodermal precursors in adherent monoculture. Nature Biotechnology 21: 183-186.

2. Ying QL, Smith AG (2003) Defined conditions for neural commitment and differentiation. Methods Enzymol 365: 327-341.

3. Smith AG (1991) Culture and differentiation of embryonic stem cells. J Tiss Cult Meth 13: 89-94.

4. Stavridis MP, Smith AG (2003) Neural differentiation of mouse embryonic stem cells. Biochem Soc Trans 31: 45-49.

5. Reynolds BA, Weiss S (1996) Clonal and population analyses demonstrate that an EGF-responsive mammalian embryonic CNS precursor is a stem cell. Dev Biol 175: 1-13.
