# Supplementary material for: Niche-Independent Symmetrical Self-Renewal of a Mammalian Tissue Stem Cell
Source: PLoS Biol. 2005 Aug 16;3(9):e283. doi: 10.1371/journal.pbio.0030283 (PMC1184591; doi:10.1371/journal.pbio.0030283)
Supplement: Supplementary file 6 [file pbio.0030283.st001.doc]

# **Supplementary Table S1**

# Table of primers used for RT-PCR

| **Gene** | **Forward primer** | **Reverse primer** | **Size (bp)** |
| --- | --- | --- | --- |
| *β-actin* | GGCCCAGAGCAAGAGAGGTATCC | ACGCACGATTTCCCTCTCAGC | 460 |
| *dlx2* | AACCACGCACCATCTACTCC | CCGCTTTTCCACATCTTCTT | 181 |
| *emx1* | GGATTCGCACAGCCTTCTC | CCCTTCCTCTTCCAGCTTCT | 600 |
| *emx2* | GCCTTTTGCAAAGGAGAGAA | GTGGGGTAAGGAAAGGAAG | 244 |
| *fabp7 (BLBP)* | GGGTAAGACCCGAGTTCCTC | ATCACCACTTTGCCACCTTC | 213 |
| *gli3* | TGGCTTGATTGTTCACGAGAGCA | TGTGTTCTGTTTTGGTGCTTGGC | 254 |
| *lhx6* | CTGAGGCAGCAGAATAGCTG | AAGCACCTTCTCCTCAACGA | 188 |
| *mash1* | CTCGTCCTCTCCGGAACTGATG | CGACAGGACGCCGCGCTGAAAG | 301 |
| *nanog* | ATGAAGTGCAAGCGGTGGCAGAAA | CCTGGTGGAGTCACAGAGTAGTTC | 464 |
| *nkx2.1* | CGCCTTACCAGGACACCAT | GCTCGAGCTCGTACACCTG | 150 |
| *olig2* | GGCGGTGGCTTCAAGTCATC | TAGTTTCGCGCCAGCAGCAG | 250 |
| *pax6* | GCTTCATCCGAGTCTTCTCCGTTAG | CCATCTTTGCTTGGGAAATCCG | 312 |
| *pax7* | AATGGCCTGTCTCCTCAGGT | TCTCCTGGCTTGATGGAGTC | 164 |
| *pou5f1 (Oct4)* | GGCGTTCTCTTTGGAAAGGTGTTC | CTCGAACCACATCCTTCTCT | 312 |
| *slc1a3 (glast)* | CCAAAAGCAACGGAGAAGAG | CCTCCCGGTAGCTCAATTTT | 229 |
| *sox1* | CCTCGGATCTCTGGTCAAGT | TACAGAGCCGGCAGTCATAC | 593 |
| *sox2* | GGCGGCAACCAGAAGAACAG | GCTTGGCCTGCGTCGATGAAC | 196 |
| *sox3* | AGACGCTGCTCAAGAAGGAC | CGTAGCCCAGCTGCTCCT | 191 |
